# Seeking central hopelessness symptoms which direct link to resilience among parents of children with autism spectrum disorder in China—A network perspective

**DOI:** 10.1002/pchj.707

**Published:** 2023-11-09

**Authors:** Yulin Huang, Yalin Huang, Miaoxuan Lin, Yanqiang Tao

**Affiliations:** ^1^ Shenzhen Jiayun Psychological Institute Shenzhen China; ^2^ Department of Neuroscience City University of Hong Kong Hong Kong China; ^3^ Division of Nutritional Sciences, School of Biosciences The University of Nottingham Leicestershire UK; ^4^ School of Psychological and Cognitive Sciences Peking University Beijing China; ^5^ Faculty of Psychology Beijing Normal University Beijing China; ^6^ Beijing Key Laboratory of Applied Experimental Psychology National Demonstration Center for Experimental Psychology Education Beijing China

**Keywords:** hopelessness, network analysis, parents with autistic children, resilience

## Abstract

The intervention process for children with autism spectrum disorder (ASD) is inextricably associated with their parents' mental health problems, such as hopelessness, which may adversely affect resilience and indirectly impact the effectiveness of interventions for their children. Hence, the motivation to help parents of children with ASD reduce hopelessness prompted us to conduct the present study and explore the interrelationship between hopelessness symptoms and resilience. This study evaluated hopelessness and resilience using the Beck Hopelessness Scale (BHS) and the Connor–Davidson Resilience Scale (CD‐RISC). Participants met the criteria for their children's ASD diagnosis by a psychiatrist (*N* = 448; 54.69% mothers; Mean_age_ = 34.59 years, *SD*_age_ = 4.94 years). Moreover, we used symptom network analysis to examine the variability in network structure between fathers and mothers. The *flow* function was applied to examine which hopelessness symptoms were directly or indirectly associated with resilience. The results showed that #BHS11 (i.e., unpleasantness‐ahead) was the central symptom found in the network structure for all parents and fathers, while #BHS17 (i.e., no‐future‐satisfaction) was the central symptom in the network structure for mothers. Additionally, #BHS6 ([NOT] expect‐to‐succeed) was directly and positively associated with resilience in all three network structures (i.e., all parents, fathers, and mothers). The results of the present study provide evidence that influential symptoms should be addressed and offer guidance for further interventions to reduce hopelessness and enhance resilience among parents of children with ASD.

## INTRODUCTION

Autism spectrum disorder (ASD) is a neurodevelopmental disorder that affects children throughout their lifetime, characterized by impaired social interactions, repetitive behaviors, and limited interests and activities (American Psychiatric Association [APA], 2013). Compared to parents of typically developing children, parents of children with ASD are more likely to have lower psychological well‐being (Banga & Ghosh, 2017), including prolonged psychological stress (Mcstay et al., 2014), anxiety (Althiabi., 2021), depression (Guller et al., 2022), and even suicidal ideation (Akram et al., 2019). The reason for this could be the constant stress of caring for their child in a challenging family and home environment (Thullen & Bonsall, 2017; Wilson & Peterson, 2018). Furthermore, individuals residing in these disadvantaged environments may also manifest neurobiological dysfunctions, notably immune inflammatory abnormalities, a factor strongly linked to suicidal ideation (Serafini et al., 2020). As a result, parents of children with ASD may develop a pessimistic attitude and experience a sense of hopelessness (Vetrayan et al., 2013). Importantly, this persistent hopelessness has been found to be correlated with resilience (Mahamid et al., 2022; Nieto et al., 2023). Therefore, parents of children with ASD should be able to identify the signs of hopelessness and take steps to enhance their resilience, thereby improving their ability to provide parent‐mediated intervention support to their children with ASD (Oono et al., 2013).

Hopelessness is commonly considered a precursor symptom of depression and may prove extremely crucial in the development of depression. It is defined by a lack of optimism and hope and is essential to address as part of the depression etiology (Sun et al., 2022). The concept of hopelessness originated from the learned helplessness theory (Seligman & Maier, 1967), which suggests that individuals may develop a sense of helplessness when they feel powerless to alter a situation, even if they try their best (Nunn & Thompson, 1996). Parents of children with ASD may experience hopelessness due to the challenges they face in caring for their children (Lawoko & Soares, 2002). This sense of hopelessness may lead to chronic sadness (Ergüner‐Tekinalp & Akkök, 2004), a phenomenon referred to as chronic sorrow (Phillips, 1991). The attributional theory of achievement motivation and emotion posits that negative emotions and expectations can be highly correlated with behavioral motivation (Weiner, 1985). Thus, it is plausible that parents' emotional state can affect their motivation for their child's intervention, particularly in parent‐mediated interventions (Akhani et al., 2021; Oono et al., 2013), which are a highly recognized model of ASD rehabilitation training (Bradshaw et al., 2017). Therefore, addressing hopelessness in parents of children with ASD is crucial for enhancing their behavioral motivation and improving the child's rehabilitation outcomes.

Traditional approaches typically assess hopelessness by calculating an overall score, such as the mean or sum score, to determine its severity (Bauer et al., 2022; Fekih‐Romdhane et al., 2020). However, these conventional methods indicate that the symptoms are locally independent and do not cause one another. In recent years, a promising statistical approach called network analysis has emerged to address this limitation in the study of psychological and psychiatric disorders (Afzali et al., 2016; Borsboom & Cramer, 2013; Ma et al., 2023; Robinaugh et al., 2020; Tao, Hou, et al., 2023). Network analysis is centered on revealing the interconnectedness of various symptoms and conceptualizing disorders as emerging from complex systems characterized by dynamic interactions among symptoms (Borsboom & Cramer, 2013). Specifically, networks are composed of nodes (depicted as circles) and edges (represented as lines), which signify an association between two nodes. In the field of psychopathology (Borsboom & Cramer, 2013; Epskamp et al., 2012), nodes typically represent symptoms, and edges denote a connection between two nodes. The thickness of an edge serves as an indicator of the strength of the association, and it can be understood as a measure of the likelihood of co‐activation. Of direct relevance to the present study is the work of Marchetti (2019) and Tao, Niu, et al. (2023), who have employed network analysis to elucidate the internal dynamics of hopelessness symptoms and their associations among the general population.

In recent years, the impact of resilience on families has also attracted widespread attention from researchers (Hadfield & Ungar, 2018; Prime et al., 2020). “Resilience” refers to an individual's adaptive process and ability in the face of severe stress events. A study conducted by Hjemdal et al. (2012) has verified that resilience is a good predictor of hopelessness after considering various external factors (i.e., stressful life events and depressive and anxiety symptoms). Additionally, Collazzoni et al. (2020) found a negative association between resilience in adverse family experiences and hopelessness in the mediation model. While the limited research mentioned above has indeed established a connection between resilience and hopelessness from a variable perspective (Collazzoni et al., 2020; Hjemdal et al., 2012) and suggests that we can enhance resilience by mitigating hopelessness through clinical strategies, no study has yet utilized a network approach to exploring the relationship between resilience and the specific symptoms (i.e., questionnaire items) of hopelessness. Meanwhile, according to Bellido‐González et al. (2019), mothers of children with disabilities (i.e., small‐for‐gestational‐age infants) have higher psychological distress than fathers but are less resilient than fathers. We will analyze fathers and mothers separately to explore whether this phenomenon also occurs in parents of children with ASD through network analysis.

Currently, there is limited understanding of the network structure of hopelessness among parents of children with ASD, particularly regarding the role of resilience. The present study seeks to explore the intrinsic structure and complex connections among hopelessness symptoms within the network of Chinese parents of children with ASD. Importantly, it is worth noting that this approach could offer a novel strategy to address the needs of individuals who may not respond adequately to antidepressant medications when dealing with both hopelessness and depression (Serafini et al., 2018). Thus, this study tested two hypotheses under three research aims:

Aim 1: Estimate the network structure of hopelessness and identify core symptoms among parents of children with ASD.

Aim 2: Compare network structures and core symptoms of hopelessness between fathers and mothers.

Hypothesis 1 Previous studies have shown that mothers tend to have higher general depression scores than fathers, both in cross‐sectional (Olsson & Hwang, 2001) and longitudinal (Barrera et al., 2012) studies. Therefore, our first hypothesis is that the network structure of mothers and fathers differs, with the mother's structure potentially exhibiting stronger internal connections.

Aim 3: Examine the direct and indirect associations between hopelessness symptoms and resilience in the network structure of all parents, as well as potential differences between fathers and mothers.

Hypothesis 2 Given that mothers of children with disabilities tend to experience higher psychological distress than fathers but are less resilient than fathers (Bellido‐González et al., 2019), our second hypothesis is that hopelessness symptoms directly or indirectly associated with resilience may vary in the network structures of fathers and mothers.

In summary, examining the associations between hopelessness symptoms and resilience may provide insights into the factors contributing to parents' ability to cope with raising a child with ASD. Furthermore, understanding the core symptoms and network structure of hopelessness can help researchers and practitioners develop more effective interventions to support parents of children with ASD.

## METHODS

### Participants

This study was conducted by the Shenzhen Jiayun Psychological Research Center in China, from August 6th to 9th, 2022, to investigate the mental health of parents with children who have ASD. The researchers sent the questionnaire via WeChat as a QR code for parents to scan and fill out. Participants accessed the questionnaire after reading the electronic informed consent by scanning the QR code using their WeChat. A total of 512 questionnaires were collected, but 64 were excluded due to missing data in the questionnaire, specifically incorrect data about age. This left 448 participants who met the entry requirements, of whom 245 mothers (54.69%), were included in this study (Mean_age_ = 34.59 years, *SD*_age_ = 4.94).

In this study, all the children of the participating parents met the diagnostic criteria for ASD and underwent the assessment process. The process involved an initial assessment by a pediatrician to understand the child's symptoms and behavior, followed by a referral to a specialist or psychologist. The specialist or psychologist conducted a series of assessments and tests to determine if ASD symptoms were present. These assessments and tests included a family and social history, observation and behavioral assessment, intelligence testing, cognitive assessment, language and social skills testing, and medical and neurological examination.

All the children diagnosed in this study received long‐term behavioral therapy, speech and occupational therapy, medication, and educational and support services. The current study was approved by the ethics committee of Beijing Normal University (Reference number: 202112220085).

### Measurements

#### Beck Hopelessness Scale

The Beck Hopelessness Scale (BHS) is a tool used to evaluate negative beliefs about the future (Beck et al., 1974). The scale comprises 20 items, with 11 negatively worded statements (e.g., “My future seems dark to me”) and nine positively worded items (e.g., “I look forward to the future with hope and enthusiasm”). Participants were asked to indicate their agreement or disagreement with each statement, which was labeled as either “true” or “false.” Scores on the scale range from zero to 20, with higher scores indicating more pronounced hopelessness. Previous research has demonstrated the reliability and validity of the BHS when utilized with Chinese adult populations (Ma et al., 2020; Zhang et al., 2015). In the current study, the scale demonstrated a Cronbach's alpha of .782.

#### Connor–Davidson Resilience Scale

The Connor–Davidson Resilience Scale (CD‐RISC) was utilized to evaluate a person's capacity to withstand trying life events (Connor, 2003). In the Chinese version, a total of 25 items on the CD‐RISC were evaluated using a 5‐point Likert scale (with 0 denoting *not true at all* and 4 denoting *true nearly all the time*) and have good reliability (Yu & Zhang, 2007). The CD‐RISC has three dimensions: resilience (i.e., you can achieve your goals), strong (i.e., able to adapt to change), and optimism (i.e., close and secure relationships) in the Chinese version (Yu & Zhang, 2007). Participants selected the option that best matched their experiences in the past month, with higher sum scores indicating greater resilience. The Cronbach's *α* of CD‐RISC in the present study was .959.

### Data analysis

#### Network structure estimation

**FIGURE 1:**
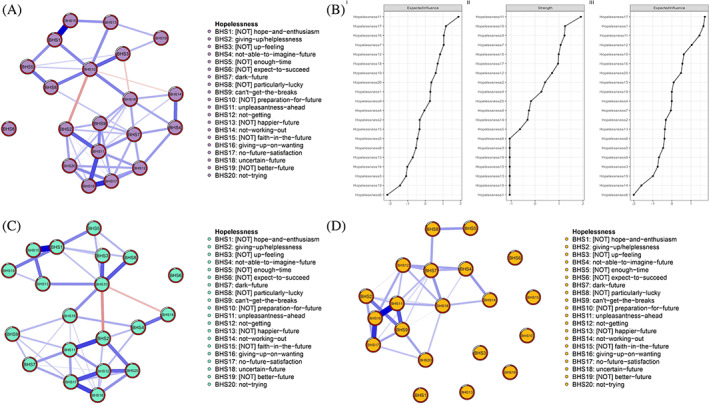
The network structure and centrality indexes. (A) All parents’ network structure. (B) The centrality of strength and expected influence (EI) (I means all parents, II means fathers, III means mothers). (C) The mothers’ network structure. (D) The fathers’ network structure.

For each BHS item, we calculated the mean, standard deviation (*SD*), skewness, and kurtosis. The Ising model is typically employed when the symptom network structures are based on binary data (van Borkulo et al., 2014). To reduce the complexity of the network structure and prevent overfitting, the enhanced least absolute shrinkage and selection operator (eLASSO) is applied, resulting in a sparse network model that is easier to comprehend compared to the original network structure (Ravikumar et al., 2010). In the network analysis, we treated each symptom of hopelessness as a “node” and the connections between these symptoms as “edges.” The thickness of the edges depicts the strength of the relationship between nodes in the network visualization. Additionally, the color of the edges indicates the partial correlation direction of the nodes. Blue and red edges represent positive and negative correlations in the symptom network (see Figure 1A,C,D), respectively. Strength is employed to indicate the degree of connectivity of a node by calculating the absolute sum of edge weights when considering all edges within the symptom structure of hopelessness. However, strength centrality may not provide an accurate prediction of node influence when the network includes negative edges (Robinaugh et al., 2016). Therefore, expected influence (EI) might be the preferred method for calculating raw edge weights that encompass both positive and negative edges. Finally, the *flow* function was employed to estimate the flow network structures between hopelessness and resilience (Epskamp et al., 2012).

The R packages “bootnet” (Epskamp et al., 2018), “networktools” (Jones, 2022), and “qgraph” (Epskamp et al., 2012) in the R program were utilized for the analysis.

#### Network stability and accuracy

Three procedures were used in this study to assess the robustness of network structures (Epskamp et al., 2018). First, the non‐parametric bootstrapping method was used to estimate confidence intervals (CIs). This method tested whether the edge weights of the sparse network model were accurate and stable. Specifically, the researchers randomly resampled the observations in the data to obtain a new dataset and compared the original observations with the new datasets to determine the accuracy of the edges. It is worth noting that narrower CIs indicate a more trustworthy network structure (Epskamp et al., 2018).

Second, the stability of the centrality indices (i.e., Strength and EI) was assessed by observing the correlation stability coefficient (*CS‐C*), which was obtained by applying subset bootstraps (Costenbader & Valente, 2003). The *CS‐C* indicated the maximum proportion of samples that could be removed, with a prerequisite that there was a 95% probability that the correlation should be at least 0.7 between the original centrality indices (Epskamp et al., 2018). Generally, the *CS‐C* should not be less than 0.25 and preferably greater than 0.5. This ensures that the centrality indices of the nodes do not change significantly after removing samples in the dataset, making the network structure's centrality indices stable.

Third, the bootstrap difference test was used to assess the network's properties (Epskamp et al., 2018), and this test relied on 95% CIs. This test was used to determine whether the weights of two edges or the centrality indices of two nodes significantly differed. The operation was performed using the R package “bootnet” (Epskamp et al., 2018).

#### Network comparison

Previous studies have shown significant differences in the mental health and resiliency of fathers and mothers (Finklestein, 2019; Russell, 2022). To investigate the differences in hopelessness network characteristics between fathers and mothers, we employed the Network Comparison Test (NCT; van Borkulo et al., 2022). The first procedure involved measuring the global network strength by comparing the absolute sum of all edge weights in the two networks. Next, we assessed the difference between the two networks based on network structure invariance. Finally, we conducted a test to compare the strength of each edge in the two networks, using a Holm–Bonferroni correction of *p*‐values. The three procedures mentioned above were carried out using the “NetworkComparisonTest” package in the R program (van Borkulo et al., 2022).

## RESULTS

### Items check and description

Based on previous studies (Mullarkey et al., 2019; Tao, Niu, et al., 2023), we first assessed item redundancy and informativeness, measured by the standard deviation of the item. We found that none of the items were redundant (i.e., the correlation between items was not statistically different by less than 25%), and all items were sufficiently informative (i.e., no item was more than 2.5 *SD*s below the mean informativeness level of 0.44 ± 0.09). As a result, all of the BHS items were included in our analyses.

Table 1 displays the mean, standard deviation, skewness, and kurtosis of hopelessness symptoms as measured by the BHS for each participant. Of all the symptoms, the highest mean rating was observed for #BHS14 (not‐working‐out), whereas #BHS6 ([NOT] expect‐to‐succeed) received the lowest mean rating.

**TABLE 1:** The basic descriptive information of BHS and CD‐RISC (*N* = 448).

Variables	Items	Symptoms	Abbreviation	*M*	*SD*	*Skewness*	*Kurtosis*
Hopelessness	1. I look forward to the future with hope and enthusiasm.	[NOT] hope‐and‐enthusiasm	BHS1	0.10	0.30	2.61	4.82
	2. I might as well give up because there's nothing I can do about making things better for myself.	Giving‐up/helplessness	BHS2	0.33	0.47	0.72	−1.49
	3. When things are going badly, I am helped by knowing they cannot stay that way forever.	[NOT] up‐feeling	BHS3	0.32	0.47	0.77	−1.41
	4. I cannot imagine what my life would be like in 10 years.	Not‐able‐to‐imagine‐future	BHS4	0.59	0.49	−0.35	−1.88
	5. I have enough time to accomplish the things I want to do.	[NOT] enough‐time	BHS5	0.25	0.43	1.18	−0.61
	6. In the future, I expect to succeed in what concerns me the most.	[NOT] expect‐to‐succeed	BHS6	0.08	0.27	3.13	7.84
	7. My future seems dark to me.	Dark‐future	BHS7	0.30	0.46	0.89	−1.22
	8. I happen to be particularly lucky, and I expect to get more of the good things in life than the average person.	[NOT] particularly‐lucky	BHS8	0.22	0.42	1.34	−0.20
	9. I just cannot get the breaks, and there's no reason I will in the future.	Cannot‐get‐the‐breaks	BHS9	0.46	0.50	0.17	−1.98
	10. My past experiences have prepared me well for the future.	[NOT] preparation‐for‐future	BHS10	0.28	0.45	0.98	−1.04
	11. All I can see ahead of me is unpleasantness rather than pleasantness.	Unpleasantness‐ahead	BHS11	0.34	0.47	0.67	−1.56
	12. I do not expect to get what I really want.	Not‐getting	BHS12	0.36	0.48	0.58	−1.66
	13. When I look ahead to the future, I expect that I will be happier than I am now.	[NOT] happier‐future	BHS13	0.15	0.36	1.96	1.84
	14. Things just do not work out the way I want them to.	Not‐working‐out	BHS14	0.68	0.47	−0.77	−1.41
	15. I have great faith in the future.	[NOT] faith‐in‐the‐future	BHS15	0.14	0.35	2.06	2.25
	16. I never get what I want, so it's foolish to want anything.	Giving‐up‐on‐wanting	BHS16	0.39	0.49	0.46	−1.80
	17. It's very unlikely that I will get any real satisfaction in the future.	No‐future‐satisfaction	BHS17	0.35	0.48	0.64	−1.60
	18. The future seems vague and uncertain to me.	Uncertain‐future	BHS18	0.48	0.50	0.09	−2.00
	19. I can look forward to more good times than bad times.	[NOT] better‐future	BHS19	0.10	0.31	2.57	4.62
	20. There's no use in really trying to get anything I want because I probably will not get it.	Not‐trying	BHS20	0.26	0.44	1.08	−0.83
Resilience			Res	2.53	0.69	0.06	−0.21

### Network structure and centrality measures analysis

The Ising model was used to assess the network of hopelessness symptoms in all parents, as shown in Figure 1A. This network consisted of 20 nodes and 190 edges, with 52 edges (27.37%) in the non‐zero edges of all parents. Several strongly correlated edges were identified, including #BHS1 ([NOT] hope‐and‐enthusiasm) – #BHS15 ([NOT] faith‐in‐the‐future), #BHS3 ([NOT] up‐feeling) – #BHS10 ([NOT] preparation‐for‐future), and #BHS11 (unpleasantness‐ahead) – #BHS16 (giving‐up‐on‐wanting) (refer to Table S1). Among these, #BHS11 (unpleasantness‐ahead) displayed the highest effective influence, followed by #BHS7 (dark‐future) and #BHS17 (no‐future‐satisfaction) (as seen in Figure 1B).

The Ising model was utilized to estimate the network of hopelessness symptoms for the mother, as shown in Figure 1C. The results were consistent with Table S2, which revealed 45 (23.68%) non‐zero edges, of which some stronger and highly correlated edges contained the following symptoms: #BHS1 ([NOT] hope‐and‐enthusiasm) – #BHS15 ([NOT] faith‐in‐the‐future), #BHS2 (giving‐up/helplessness) – #BHS11 (unpleasantness‐ahead), and #BHS8 ([NOT] particularly‐lucky) – #BHS10 ([NOT] preparation‐for‐future). Furthermore, Figure 1B indicates that #BHS17 (no‐future‐satisfaction) exhibited the highest effective influence, followed by #BHS11 (unpleasantness‐ahead), #BHS1 ([NOT] hope‐and‐enthusiasm), and #BHS12 (not‐getting).

The network structure of fathers' hopelessness was estimated separately and is shown in Figure 1D. Of the 29 (15.26%) non‐zero edges in fathers, several strongly correlated edges were identified, including #BHS16 (giving‐up‐on‐wanting) – #BHS11 (unpleasantness‐ahead), #BHS16 (giving‐up‐on‐wanting) – #BHS17 (no‐future‐satisfaction), and #BHS9 (cannot‐get‐the‐breaks) – #BHS12 (not‐getting). These findings were consistent with Table S3. Furthermore, the highest strength was observed in #BHS11 (unpleasantness‐ahead) in Figure 1 (Part B), followed by #BHS7 (dark‐future), #BHS9 (cannot‐get‐the‐breaks), and #BHS16 (giving‐up‐on‐wanting).

**FIGURE 2:**
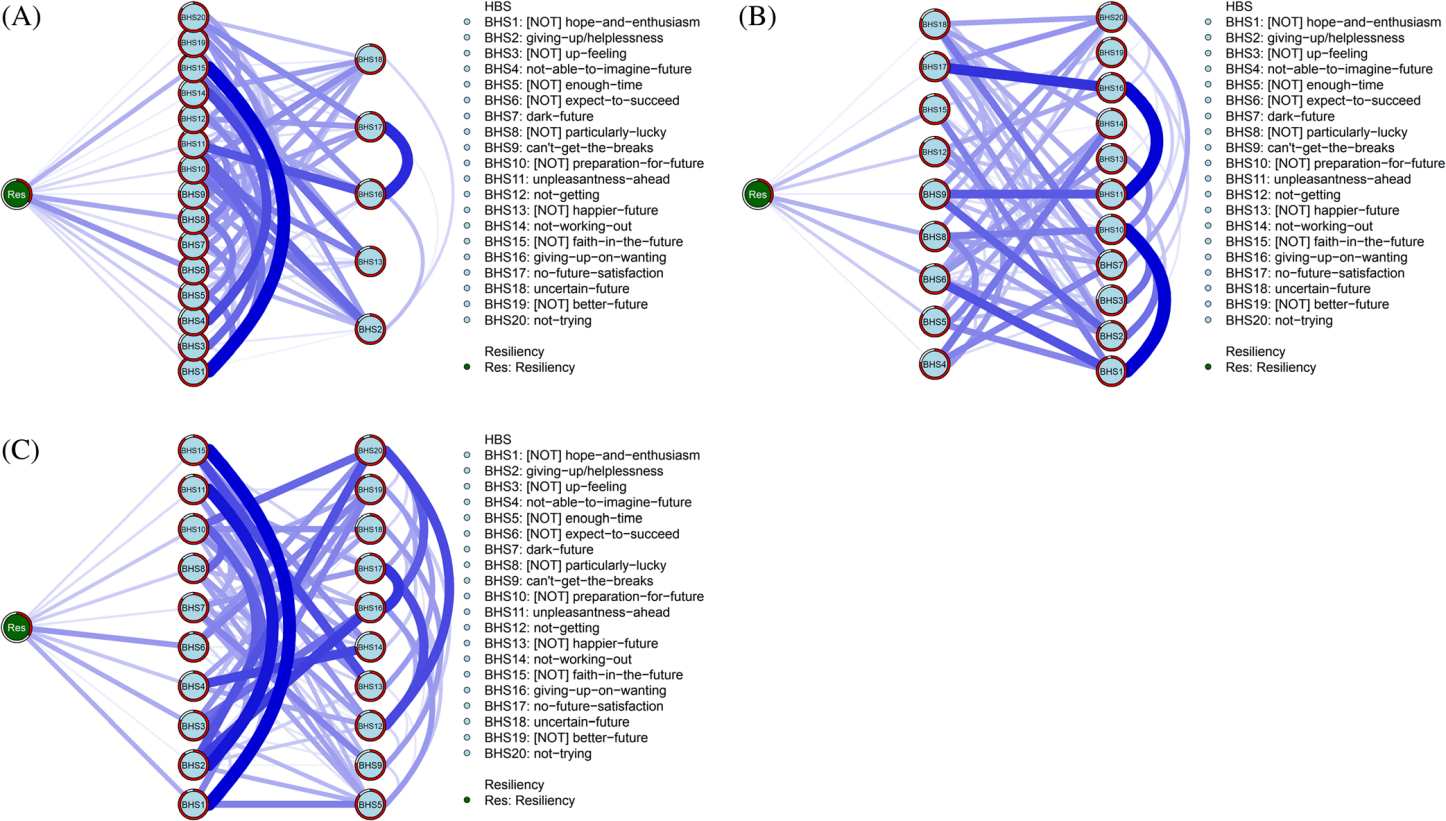
Flow network of resilience. (A) All parents’ network structure. (B) Fathers’ flow structure. (C) Mothers’ flow structure.

Based on the *flow* function analysis, we have concluded that #BHS6, #BHS8, and #BHS15 were directly related to resiliency in both parents and fathers. On the other hand, we found that #BHS1, #BHS3, and #BHS6 were directly related to resiliency among mothers (Figure 2).

### Network accuracy and stability

To ensure stability, we conducted a bootstrapped analysis and found that the 95% CIs were narrow for all parents, fathers, and mothers (see Figure S1). We also used a case‐dropping subset sample bootstrap procedure and found that the strength or *EI* reported higher stability for all parents, fathers, and mothers (i.e., *CS‐C* = .52; *CS‐C* = .36; *CS‐C* = .20; see Figure S2). These results indicate that the centrality index used in this study is interpretable.

Regarding strength, we found that #BHS10 was statistically stronger than the other symptoms in the whole network structure for all parents (Figure S3A). Similarly, in the mother's network structure, #BHS11, #BHS17, and #BHS7 were statistically stronger than the other symptoms (Figure S3B). However, in the father's network structure, we did not find any nodes that were statistically different from each other (Figure S3C). It is worth noting that the bootstrapped difference tests also revealed that a relatively large proportion of the comparisons among edge weights were statistically significant for the network structures of all parents, fathers, and mothers (Figure S4).

### Network comparison between father and mother

**FIGURE 3:**
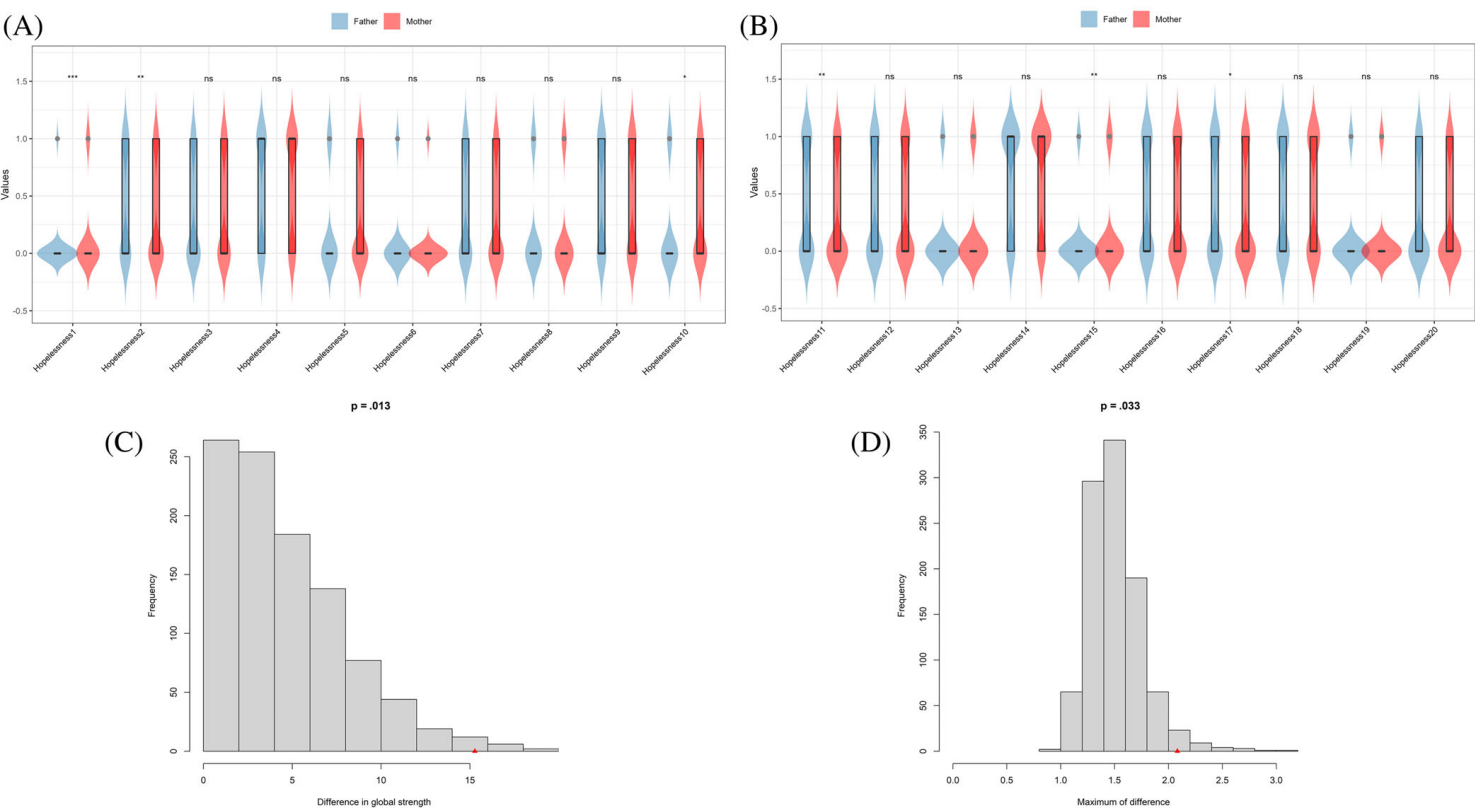
Network comparison between fathers and mothers. (A and B) Mean scores on items between fathers and mothers. (C) Global strength. (D) Edge weights.

In terms of mean levels (as illustrated in Figure 3A,B), we observed that certain hopelessness symptoms, such as #BHS10 and #BHS15, were significantly more elevated in mothers compared to fathers. Conversely, the levels of #BHS1, #BHS2, #BHS11, and #BHS17 were significantly higher in fathers than in mothers.

We compared network models and network centrality indices of fathers (*N* = 203) and mothers (*N* = 245). Our findings indicate a significant difference between the global network strength of fathers (18.42) and mothers (33.72), with a global strength difference (*S*) of 15.30 and a *p*‐value of .013 **(**see Figure 3C). In the specific symptom centrality test, we discovered that the EI value of #BHS1 was significantly higher in mothers compared to fathers (*p* < .05). Additionally, we found a significant maximum difference in edge weights of the observed networks (*M* = 2.08, *p* = .033) between the two groups (see Figure 3D). However, there was no significant difference in individual edge weights between fathers and mothers (all *p* values >.05 after Holm–Bonferroni corrections). Therefore, caution is necessary in drawing conclusions from this study.

## DISCUSSION

The present study examines the network structure of hopelessness and identifies central symptoms among parents of children with ASD. It was found that among all parents and fathers, the most central symptom was “unpleasantness‐ahead” (#BHS11), whereas in the network of mothers, the central symptom was “no‐future‐satisfaction” (#BHS17). Additionally, the study determines which symptoms are more strongly associated with resilience using a network analysis approach. Specifically, #BHS6 ([NOT] expect‐to‐succeed) is consistently linked to resilience in all three networks.

The findings of our research on central symptoms and their stronger association with resilience, using a network analysis approach, support the initial hypothesis that there are gender differences in the network structure and global strength of symptoms. One potential explanation for this difference is that mothers and fathers employ different coping strategies to deal with their children's problem behavior (Al‐Yagon et al., 2023; Hastings, Kovshoff, Brown et al., 2005; Rattaz et al., 2023). Specifically, Al‐Oran et al. (2022) demonstrated that mothers tend to use more problem‐focused and emotion‐focused coping strategies than fathers. Additionally, mothers are generally more socially engaged with their children than fathers (Ozturk et al., 2014). Hence, the lack of effective treatment for ASD may lead to pessimism among mothers regarding the future, resulting in the no‐future‐satisfaction symptom (#BHS17).

In contrast, Hastings (2003) reported that fathers' stress is not associated with their children's behavior but is positively correlated with the mother's depression score (Hastings, Kovshoff, Ward et al., 2005). This supports the evidence that fathers experience “unpleasantness‐ahead” (#BHS11) in their own circumstances. King (2008) suggested that negative thoughts, strongly associated with hopelessness, are central symptoms that psychotherapists can target to help patients interpret unpleasant experiences positively and reduce hopelessness (i.e., #BHS11). Cognitive behavioral therapy can effectively improve negative thought patterns and eliminate undesirable emotions (Kaczkurkin & Foa, 2015; Weiss et al., 2018). While centrality indicators may not always equate to clinical relevance, they still provide psychotherapists with a good direction for intervention, as they are the causal endpoint of many network pathways (Fried et al., 2018).

The findings indicate that #BHS1 ([NOT] hope‐and‐enthusiasm) and #BHS15 ([NOT] faith‐in‐the‐future) exhibit the most noteworthy association among all the parents in the network structure. Losing faith in the future is accompanied by a loss of hope and enthusiasm. Optimism, which emphasizes believing in outcomes (Ciarrocchi et al., 2008), can help parents believe in their competence to ensure a successful and satisfying future for their families and children (Alarcon et al., 2013). However, interventions for ASD may not always yield ideal results, leading parents to struggle with maintaining their optimism. Thus, it becomes imperative to enhance psychological capital, which includes self‐confidence, optimism, and hope, for both mothers and fathers to maintain a stable personality and belief in a prosperous and positive future (Alarcon et al., 2013). The aforementioned attributes enhance the well‐being of parents and promote a positive familial environment that nurtures the growth and development of children (Xiao et al., 2022).

The strongest correlation edge in the network structure of fathers was between #BHS11 (unpleasantness‐ahead) and #BHS16 (giving‐up‐on‐wanting). Fathers who reported being unable to obtain what they wanted also reported experiencing more unpleasantness. Externalize theory and desire theory suggest that pleasantness or unpleasantness can be external to the sensation and may relate to feeling desired or unwanted (Aydede, 2014). The fact that #BHS11 (unpleasantness‐ahead) was a central symptom in the network of fathers indicates that unmet desires may be the primary reason for this symptom. In the network structure of mothers, #BHS2 (giving up/helplessness) and #BHS11 (unpleasantness‐ahead) were highly correlated, reflecting frustration theory (Brown & Farber, 1951), which suggests that negative emotions and feelings of helplessness can arise when motivation is hindered and individual needs are unmet. Research by Wade et al. (1990) supports this theory, as they found that anxiety and frustration can predict unpleasantness. Mothers of children with ASD face significant challenges, and prolonged exposure to frustration may lead to a loss of motivation and giving up. For example, difficulties with social interactions can make it challenging for mothers to communicate effectively, even leading to unresponsiveness with their autistic children, and may decrease motivation to foster good parent–child communication (Zlomke & Jeter, 2020).

In our final analysis, we explored the relationship between hopelessness and resilience within the network using a flow function. Our results revealed that 10 symptoms were directly linked to resilience in the network structure of mothers, with #BHS1, #BHS3, and #BHS6 showing strong connections to resilience. Compared to fathers, mothers tend to provide more direct care to their children (Hastings, Kovshoff, Brown, et al., 2005; Shattnawi et al., 2020), which can be challenging and unpredictable due to the lack of social policies. This situation may lead mothers to experience feelings of helplessness. However, it can also contribute to the development of their ability to cope with frustration (Dor‐Shav & Mikulincer, 1990; Rosellini & Seligman, 1975). In the network structure of fathers, we identified nine hopelessness symptoms that were directly related to resilience, with #BHS6, #BHS8, and #BHS15 exhibiting strong connections to resilience. Notably, most fathers are responsible for the family's finances, while mothers are responsible for the direct care of the children (Bachem et al., 2018; Ball et al., 1996). This difference in roles may explain why different symptoms in the flow network structure affect the resilience of fathers and mothers differently. Additionally, according to Brody and Simmons (2007), fathers tend to demonstrate greater resilience in coping with changes in the family.

Interestingly, our network analysis of hopelessness revealed some independent symptoms, particularly #BHS6 ([NOT] expect‐to‐succeed), which were unrelated to the other symptoms and strongly associated with resilience in all three network structures. This finding suggests that parental resilience may be stronger if a parent does not have high expectations of success. It also supports the idea that resilience can be strengthened when faced with challenges and adversity (Margalit & Kleitman, 2006). Furthermore, intervention can facilitate and strengthen resilience for anyone (Hartling, 2008). Our study aims to provide guidance for interventions to reduce parental hopelessness and enhance resilience.

### Limitations

Several limitations must be considered when interpreting the results and implications of this study. First, it is a cross‐sectional study, and cohort effects may exist. The level of hopelessness reported may be influenced by the current social context, given that the study was conducted during the COVID‐19 pandemic. The impact of the epidemic may have created additional challenges for parents and children in terms of living situations and interventions. Second, the study was conducted among parents from a single institution, with more than half of the parents from the local area (Guangdong Province). Therefore, the results may not fully reflect the diversity of parents of children with ASD across different regions. The patterns and clinical characteristics of hopelessness are closely related to social support (e.g., government policies) and economic background among parents of children with ASD. Economic development and government policies vary across regions, and future studies should control for the number of subjects from different cities. Lastly, the study did not collect information on the timing of the children's ASD diagnosis or when they received interventions, rehabilitation treatments, and so forth. This limits our ability to explain parental resilience in the face of adversity. Furthermore, our research does not provide evidence regarding the clinical efficacy of interventions aimed at enhancing resilience or their impact on the recovery outcomes of children with ASD.

### Implications and further research

Based on our research, we have confirmed significant differences and identified central symptom nodes of hopelessness among parents of children with ASD. Consequently, we can tailor clinical interventions for parents based on different symptom nodes, with the goal of preventing the progression of their hopelessness to an irreversible state. For future studies, it is recommended to incorporate information about the children, such as the time of diagnosis and the severity of the condition, as part of auxiliary analyses. To enhance the clinical significance of the research, further analysis could be conducted in longitudinal studies to investigate whether adjusting parental resilience positively, using different intervention strategies, impacts both the parents' hopelessness and the recovery outcomes of children with ASD.

### Conclusion

This study used network analysis to investigate the structure of hopelessness symptoms in parents of children with ASD. The results revealed significant differences between fathers and mothers, particularly in terms of central symptoms. For all parents and fathers, the central symptom identified in the network structure was #BHS11 (unpleasantness‐ahead), while for mothers, it was #BHS17 (no‐future‐satisfaction). Moreover, #BHS6 ([NOT] expect‐to‐succeed) was found to be strongly associated with resilience in all three network structures (i.e., fathers, mothers, and all parents).

## CONFLICT OF INTEREST STATEMENT

The authors declare no conflicts of interest.

## ETHICS STATEMENT

The study was approved by the University of Beijing Normal University Institutional Review Board (202112220085).

## Supporting information


**Table S1.** The weight‐matrix among all parents.
**Table S2.** The weight‐matrix among mothers.
**Table S3.** The weight‐matrix among fathers.
**Figure S1.** Nonparametric bootstrapped confidence intervals of estimated edges. The red line represents the estimated edge, while the dark area indicates the 95% bootstrap confidence interval. (A) Indicates all parents. (B) Indicates fathers. (C) Indicates mothers.
**Figure S2.** The x‐axis indicates the percentage of cases of the original sample included at each step. The y‐axis indicates the average correlations between the original network's centrality indices and the centrality indices from the networks that were re‐estimated after excluding increasing percentages of cases. (A) Indicates all parents. (B) Indicates fathers. (C) Indicates mothers.
**Figure S3.** Bootstrapped stability test for edge‐weight. The results of the bootstrapped difference tests (α = .05) for edge‐weights are shown in this figure. The color of the boxes indicates whether edge‐weights differ significantly from each other (i.e., black) or do not differ significantly (i.e., grey). (A) Indicates all parents. (B) Indicates fathers. (C) Indicates mothers.
**Figure S4.** Bootstrapped stability test for edge‐weight. The results of the bootstrapped difference tests (α = .05) for edge‐weights are shown in this figure. The color of the boxes indicates whether edge‐weights differ significantly from each other (i.e., black) or do not differ significantly (i.e., grey). The diagonal line indicates the strength of edge‐weights, shifting from red (negative associations) to white (representing weaker edges) and ultimately blue (representing stronger edge‐weights). (A) Indicates all parents. (B) Indicates fathers. (C) Indicates mothers.

## Data Availability

Data are available upon request from the first author.
