# Supplementary material for: Seeking central hopelessness symptoms which direct link to resilience among parents of children with autism spectrum disorder in China—A network perspective
Source: Psych J. 2023 Nov 9;13(1):102–12. doi: 10.1002/pchj.707 (PMC10917102; doi:10.1002/pchj.707)
Supplement: Supplementary file 1 — Table S1. The weight‐matrix among all parents. Table S2. The weight‐matrix among mothers. Table S3. The weight‐matrix among fathers. Figure S1. Nonparametric bootstrapped confidence intervals of estimated edges. The red line represents the estimated edge, while the dark area indicates the 95% bootstrap confidence interval. (A) Indicates all parents. (B) Indicates fathers. (C) Indicates mothers. Figure S2. The x‐axis indicates the percentage of cases of the original sample included at each step. The y‐axis indicates the average correlations between the original network's centrality indices and the centrality indices from the networks that were re‐estimated after excluding increasing percentages of cases. (A) Indicates all parents. (B) Indicates fathers. (C) Indicates mothers. Figure S3. Bootstrapped stability test for edge‐weight. The results of the bootstrapped difference tests (α = .05) for edge‐weights are shown in this figure. The color of the boxes indicates whether edge‐weights differ significantly from each other (i.e., black) or do not differ significantly (i.e., grey). (A) Indicates all parents. (B) Indicates fathers. (C) Indicates mothers. Figure S4. Bootstrapped stability test for edge‐weight. The results of the bootstrapped difference tests (α = .05) for edge‐weights are shown in this figure. The color of the boxes indicates whether edge‐weights differ significantly from each other (i.e., black) or do not differ significantly (i.e., grey). The diagonal line indicates the strength of edge‐weights, shifting from red (negative associations) to white (representing weaker edges) and ultimately blue (representing stronger edge‐weights). (A) Indicates all parents. (B) Indicates fathers. (C) Indicates mothers. [file PCHJ-13-102-s001.docx]

Table S1. The weight-matrix among all parents.

|  | 1 | 2 | 3 | 4 | 5 | 6 | 7 | 8 | 9 | 10 | 11 | 12 | 13 | 14 | 15 | 16 | 17 | 18 | 19 | 20 |
| --- | --- | --- | --- | --- | --- | --- | --- | --- | --- | --- | --- | --- | --- | --- | --- | --- | --- | --- | --- | --- |
| 1 Hopelessness1 | 0 | 0 | 0 | 0 | 0.797 | 0 | 0 | 0 | 0 | 0.878 | 0 | 0 | 0 | 0 | 1.891 | 0 | 0 | 0 | 0 | 0 |
| 2 Hopelessness2 | 0 | 0 | 0 | 0 | 0 | 0 | 0.456 | 0 | 0.569 | -0.720 | 1.165 | 0 | 0 | 0 | 0 | 0.373 | 0.163 | 0 | 0 | 0.679 |
| 3 Hopelessness3 | 0 | 0 | 0 | 0 | 0.304 | 0 | 0 | 0 | 0 | 1.128 | 0 | 0 | 0 | 0 | 0 | 0 | 0 | -0.395 | 0.525 | 0 |
| 4 Hopelessness4 | 0 | 0 | 0 | 0 | 0 | 0 | 0.659 | 0 | 0 | 0 | 0 | 0.725 | 0 | 1.062 | 0 | 0 | 0 | 0.628 | 0 | 0 |
| 5 Hopelessness5 | 0.797 | 0 | 0.304 | 0 | 0 | 0 | 0 | 1.021 | 0 | 0.373 | 0 | 0 | 0 | 0 | 0 | 0 | 0 | 0 | 0 | 0 |
| 6 Hopelessness6 | 0 | 0 | 0 | 0 | 0 | 0 | 0 | 0 | 0 | 0 | 0 | 0 | 0 | 0 | 0 | 0 | 0 | 0 | 0 | 0 |
| 7 Hopelessness7 | 0 | 0.456 | 0 | 0.659 | 0 | 0 | 0 | 0.405 | 0.571 | 0 | 0.758 | 0.621 | 0 | 0 | 0 | 0 | 0.585 | 0.620 | 0 | 0 |
| 8 Hopelessness8 | 0 | 0 | 0 | 0 | 1.021 | 0 | 0.405 | 0 | 0 | 0.953 | 0 | 0 | 0 | 0 | 0 | 0 | 0 | 0 | 0 | 0 |
| 9 Hopelessness9 | 0 | 0.569 | 0 | 0 | 0 | 0 | 0.571 | 0 | 0 | 0 | 0.919 | 0 | 0 | 0 | 0 | 0 | 0.676 | 0.594 | 0 | 0.208 |
| 10 Hopelessness10 | 0.878 | -0.720 | 1.128 | 0 | 0.373 | 0 | 0 | 0.953 | 0 | 0 | 0 | 0 | 0.800 | -0.300 | 0 | 0 | 0 | 0.902 | 0 | 0 |
| 11 Hopelessness11 | 0 | 1.165 | 0 | 0 | 0 | 0 | 0.758 | 0 | 0.919 | 0 | 0 | 0.705 | 0 | 0.331 | 0 | 1.311 | 0.282 | 0.373 | 0 | 0.057 |
| 12 Hopelessness12 | 0 | 0 | 0 | 0.725 | 0 | 0 | 0.621 | 0 | 0 | 0 | 0.705 | 0 | 0 | 0 | 0 | 0.635 | 0.761 | 0.282 | 0 | 0.647 |
| 13 Hopelessness13 | 0 | 0 | 0 | 0 | 0 | 0 | 0 | 0 | 0 | 0.800 | 0 | 0 | 0 | 0 | 0.781 | 0 | 0 | 0 | 0.531 | 0 |
| 14 Hopelessness14 | 0 | 0 | 0 | 1.062 | 0 | 0 | 0 | 0 | 0 | -0.300 | 0.331 | 0 | 0 | 0 | 0 | 0 | 0 | 0.565 | 0 | 0 |
| 15 Hopelessness15 | 1.891 | 0 | 0 | 0 | 0 | 0 | 0 | 0 | 0 | 0 | 0 | 0 | 0.781 | 0 | 0 | 0 | 0 | 0 | 0 | 0 |
| 16 Hopelessness16 | 0 | 0.373 | 0 | 0 | 0 | 0 | 0 | 0 | 0 | 0 | 1.311 | 0.635 | 0 | 0 | 0 | 0 | 1.541 | 0.110 | 0 | 0.712 |
| 17 Hopelessness17 | 0 | 0.163 | 0 | 0 | 0 | 0 | 0.585 | 0 | 0.676 | 0 | 0.282 | 0.761 | 0 | 0 | 0 | 1.541 | 0 | 0 | 0 | 0.852 |
| 18 Hopelessness18 | 0 | 0 | -0.395 | 0.628 | 0 | 0 | 0.620 | 0 | 0.594 | 0.902 | 0.373 | 0.282 | 0 | 0.565 | 0 | 0.110 | 0 | 0 | 0 | 0.496 |
| 19 Hopelessness19 | 0 | 0 | 0.525 | 0 | 0 | 0 | 0 | 0 | 0 | 0 | 0 | 0 | 0.531 | 0 | 0 | 0 | 0 | 0 | 0 | 0 |
| 20 Hopelessness20 | 0 | 0.679 | 0 | 0 | 0 | 0 | 0 | 0 | 0.208 | 0 | 0.057 | 0.647 | 0 | 0 | 0 | 0.712 | 0.852 | 0.496 | 0 | 0 |

Table S2. The weight-matrix among mother.

|  | 1 | 2 | 3 | 4 | 5 | 6 | 7 | 8 | 9 | 10 | 11 | 12 | 13 | 14 | 15 | 16 | 17 | 18 | 19 | 20 |
| --- | --- | --- | --- | --- | --- | --- | --- | --- | --- | --- | --- | --- | --- | --- | --- | --- | --- | --- | --- | --- |
| 1 Hopelessness1 | 0 | 0 | 0.451 | 0 | 0.983 | 0 | 0 | 0.554 | 0 | 0 | 0 | 0.451 | 0 | 0.983 | 0 | 0 | 0.554 | 0 | 0 | 0 |
| 2 Hopelessness2 | 0 | 0 | 0 | 0 | 0 | 0 | 0.284 | 0 | 0 | 0 | 0 | 0 | 0 | 0 | 0 | 0.284 | 0 | 0 | 0 | 0 |
| 3 Hopelessness3 | 0.451 | 0 | 0 | 0 | 0.186 | 0 | 0 | 0 | 0 | 0.451 | 0 | 0 | 0 | 0.186 | 0 | 0 | 0 | 0 | 0.451 | 0 |
| 4 Hopelessness4 | 0 | 0 | 0 | 0 | 0 | 0 | 0.137 | 0 | 0 | 0 | 0 | 0 | 0 | 0 | 0 | 0.137 | 0 | 0 | 0 | 0 |
| 5 Hopelessness5 | 0.983 | 0 | 0.186 | 0 | 0 | 0 | 0 | 0.604 | 0 | 0.983 | 0 | 0.186 | 0 | 0 | 0 | 0 | 0.604 | 0 | 0.983 | 0 |
| 6 Hopelessness6 | 0 | 0 | 0 | 0 | 0 | 0 | 0 | 0 | 0 | 0 | 0 | 0 | 0 | 0 | 0 | 0 | 0 | 0 | 0 | 0 |
| 7 Hopelessness7 | 0 | 0.284 | 0 | 0.137 | 0 | 0 | 0 | 0 | 0.523 | 0 | 0.284 | 0 | 0.137 | 0 | 0 | 0 | 0 | 0.523 | 0 | 0.284 |
| 8 Hopelessness8 | 0.554 | 0 | 0 | 0 | 0.604 | 0 | 0 | 0 | 0 | 0.554 | 0 | 0 | 0 | 0.604 | 0 | 0 | 0 | 0 | 0.554 | 0 |
| 9 Hopelessness9 | 0 | 0 | 0 | 0 | 0 | 0 | 0.523 | 0 | 0 | 0 | 0 | 0 | 0 | 0 | 0 | 0.523 | 0 | 0 | 0 | 0 |
| 10 Hopelessness10 | 0.478 | -0.890 | 1.295 | 0 | 0.599 | 0 | 0 | 1.326 | 0 | 0.478 | -0.890 | 1.295 | 0 | 0.599 | 0 | 0 | 1.326 | 0 | 0.478 | -0.890 |
| 11 Hopelessness11 | 0 | 1.537 | 0 | 0.543 | 0 | 0 | 0.828 | 0 | 0.528 | 0 | 1.537 | 0 | 0.543 | 0 | 0 | 0.828 | 0 | 0.528 | 0 | 1.537 |
| 12 Hopelessness12 | 0 | 0 | 0 | 0.546 | 0 | 0 | 0.211 | 0 | 0 | 0 | 0 | 0 | 0.546 | 0 | 0 | 0.211 | 0 | 0 | 0 | 0 |
| 13 Hopelessness13 | 0 | 0 | 0 | 0 | 0 | 0 | 0 | 0 | 0 | 0 | 0 | 0 | 0 | 0 | 0 | 0 | 0 | 0 | 0 | 0 |
| 14 Hopelessness14 | 0 | 0 | 0 | 1.170 | 0 | 0 | 0 | 0 | 0 | 0 | 0 | 0 | 1.170 | 0 | 0 | 0 | 0 | 0 | 0 | 0 |
| 15 Hopelessness15 | 2.082 | 0 | 0 | 0 | 0 | 0 | 0 | 0 | 0 | 2.082 | 0 | 0 | 0 | 0 | 0 | 0 | 0 | 0 | 2.082 | 0 |
| 16 Hopelessness16 | 0 | 0.526 | 0 | 0 | 0 | 0 | 0 | 0 | 0 | 0 | 0.526 | 0 | 0 | 0 | 0 | 0 | 0 | 0 | 0 | 0.526 |
| 17 Hopelessness17 | 0 | 0 | 0 | 0 | 0 | 0 | 0.693 | 0 | 0.413 | 0 | 0 | 0 | 0 | 0 | 0 | 0.693 | 0 | 0.413 | 0 | 0 |
| 18 Hopelessness18 | 0 | 0 | 0 | 0.673 | 0 | 0 | 0.320 | 0 | 0.571 | 0 | 0 | 0 | 0.673 | 0 | 0 | 0.320 | 0 | 0.571 | 0 | 0 |
| 19 Hopelessness19 | 1.072 | 0 | 0 | 0 | 0 | 0 | 0 | 0 | 0 | 1.072 | 0 | 0 | 0 | 0 | 0 | 0 | 0 | 0 | 1.072 | 0 |
| 20 Hopelessness20 | 0 | 1.132 | 0 | 0 | 0 | 0 | 0 | 0 | 0 | 0 | 1.132 | 0 | 0 | 0 | 0 | 0 | 0 | 0 | 0 | 1.132 |

Table S3. The weight-matrix among father

|  | 1 | 2 | 3 | 4 | 5 | 6 | 7 | 8 | 9 | 10 | 11 | 12 | 13 | 14 | 15 | 16 | 17 | 18 | 19 | 20 |
| --- | --- | --- | --- | --- | --- | --- | --- | --- | --- | --- | --- | --- | --- | --- | --- | --- | --- | --- | --- | --- |
| 1 Hopelessness1 | 0 | 0 | 0 | 0 | 0 | 0 | 0 | 0 | 0 | 0 | 0 | 0 | 0 | 0 | 0 | 0 | 0 | 0 | 0 | 0 |
| 2 Hopelessness2 | 0 | 0 | 0 | 0 | 0 | 0 | 0 | 0 | 1.007 | 0 | 0 | 0 | 0 | 0 | 0 | 0 | 0 | 0 | 1.007 | 0 |
| 3 Hopelessness3 | 0 | 0 | 0 | 0 | 0 | 0 | 0 | 0 | 0 | 0 | 0 | 0 | 0 | 0 | 0 | 0 | 0 | 0 | 0 | 0 |
| 4 Hopelessness4 | 0 | 0 | 0 | 0 | 0 | 0 | 0.582 | 0 | 0 | 0 | 0 | 0 | 0 | 0 | 0 | 0 | 0.582 | 0 | 0 | 0 |
| 5 Hopelessness5 | 0 | 0 | 0 | 0 | 0 | 0 | 0 | 0.741 | 0 | 0 | 0 | 0 | 0 | 0 | 0 | 0 | 0 | 0.741 | 0 | 0 |
| 6 Hopelessness6 | 0 | 0 | 0 | 0 | 0 | 0 | 0 | 0 | 0 | 0 | 0 | 0 | 0 | 0 | 0 | 0 | 0 | 0 | 0 | 0 |
| 7 Hopelessness7 | 0 | 0 | 0 | 0.582 | 0 | 0 | 0 | 0.655 | 0.319 | 0 | 0 | 0 | 0 | 0.582 | 0 | 0 | 0 | 0.655 | 0.319 | 0 |
| 8 Hopelessness8 | 0 | 0 | 0 | 0 | 0.741 | 0 | 0.655 | 0 | 0 | 0 | 0 | 0 | 0 | 0 | 0.741 | 0 | 0.655 | 0 | 0 | 0 |
| 9 Hopelessness9 | 0 | 1.007 | 0 | 0 | 0 | 0 | 0.319 | 0 | 0 | 0 | 0 | 1.007 | 0 | 0 | 0 | 0 | 0.319 | 0 | 0 | 0 |
| 10 Hopelessness10 | 0 | 0 | 0 | 0 | 0 | 0 | 0 | 0 | 0 | 0 | 0 | 0 | 0 | 0 | 0 | 0 | 0 | 0 | 0 | 0 |
| 11 Hopelessness11 | 0 | 0.495 | 0 | 0 | 0 | 0 | 0.467 | 0 | 1.252 | 0 | 0 | 0.495 | 0 | 0 | 0 | 0 | 0.467 | 0 | 1.252 | 0 |
| 12 Hopelessness12 | 0 | 0.125 | 0 | 0.675 | 0 | 0 | 0.631 | 0 | 0 | 0 | 0 | 0.125 | 0 | 0.675 | 0 | 0 | 0.631 | 0 | 0 | 0 |
| 13 Hopelessness13 | 0 | 0 | 0 | 0 | 0 | 0 | 0 | 0 | 0 | 0 | 0 | 0 | 0 | 0 | 0 | 0 | 0 | 0 | 0 | 0 |
| 14 Hopelessness14 | 0 | 0 | 0 | 0.746 | 0 | 0 | 0 | 0 | 0 | 0 | 0 | 0 | 0 | 0.746 | 0 | 0 | 0 | 0 | 0 | 0 |
| 15 Hopelessness15 | 0 | 0 | 0 | 0 | 0 | 0 | 0 | 0 | 0 | 0 | 0 | 0 | 0 | 0 | 0 | 0 | 0 | 0 | 0 | 0 |
| 16 Hopelessness16 | 0 | 0 | 0 | 0 | 0 | 0 | 0 | 0 | 0 | 0 | 0 | 0 | 0 | 0 | 0 | 0 | 0 | 0 | 0 | 0 |
| 17 Hopelessness17 | 0 | 0.651 | 0 | 0 | 0 | 0 | 0.269 | 0 | 0.633 | 0 | 0 | 0.651 | 0 | 0 | 0 | 0 | 0.269 | 0 | 0.633 | 0 |
| 18 Hopelessness18 | 0 | 0.285 | 0 | 0.290 | 0 | 0 | 0.769 | 0 | 0.098 | 0 | 0 | 0.285 | 0 | 0.290 | 0 | 0 | 0.769 | 0 | 0.098 | 0 |
| 19 Hopelessness19 | 0 | 0 | 0 | 0 | 0 | 0 | 0 | 0 | 0 | 0 | 0 | 0 | 0 | 0 | 0 | 0 | 0 | 0 | 0 | 0 |
| 20 Hopelessness20 | 0 | 0 | 0 | 0 | 0 | 0 | 0 | 0 | 0.665 | 0 | 0 | 0 | 0 | 0 | 0 | 0 | 0 | 0 | 0.665 | 0 |


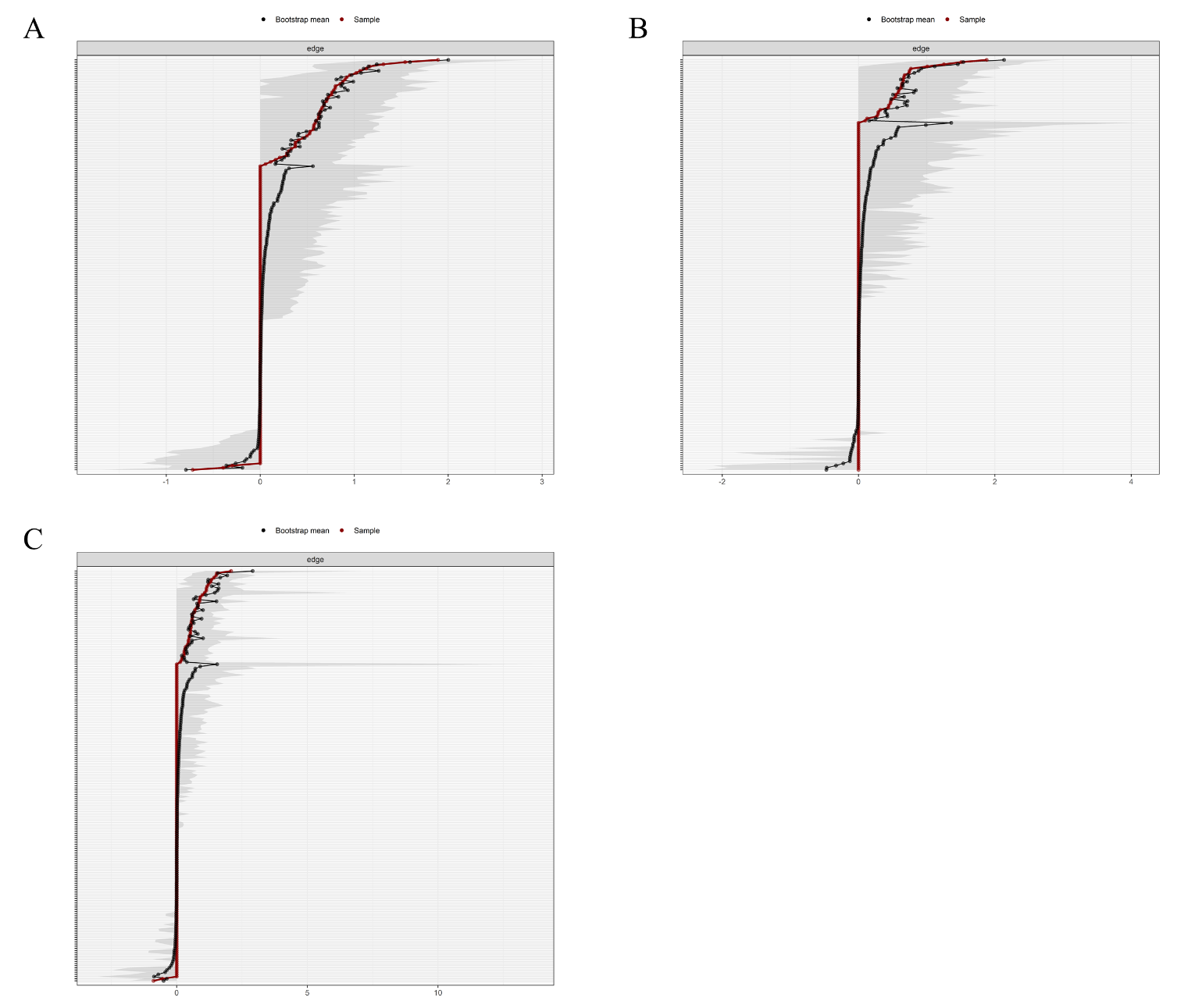


**Figure S1.** Nonparametric bootstrapped confidence intervals of estimated edges. The red line represents the estimated edge, while the dark area indicates the 95% bootstrap confidence interval. A indicates all parents. B indicates fathers. C indicates mothers.


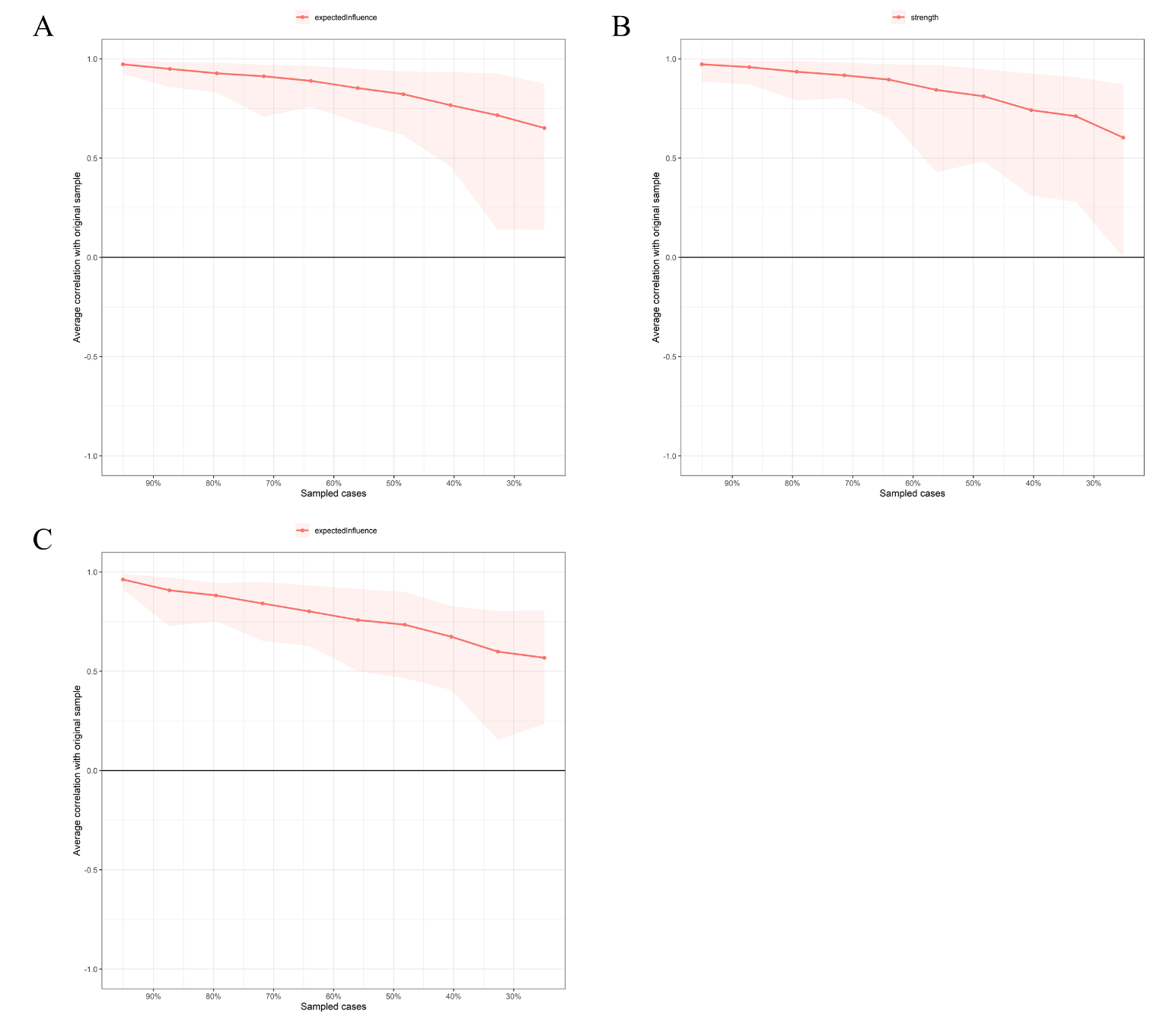


Figure S2. The x-axis indicates the percentage of cases of the original sample included at each step. The y-axis indicates the average correlations between the original network's centrality indices and the centrality indices from the networks that were re-estimated after excluding increasing percentages of cases. A indicates all parents. B indicates fathers. C indicates mothers.


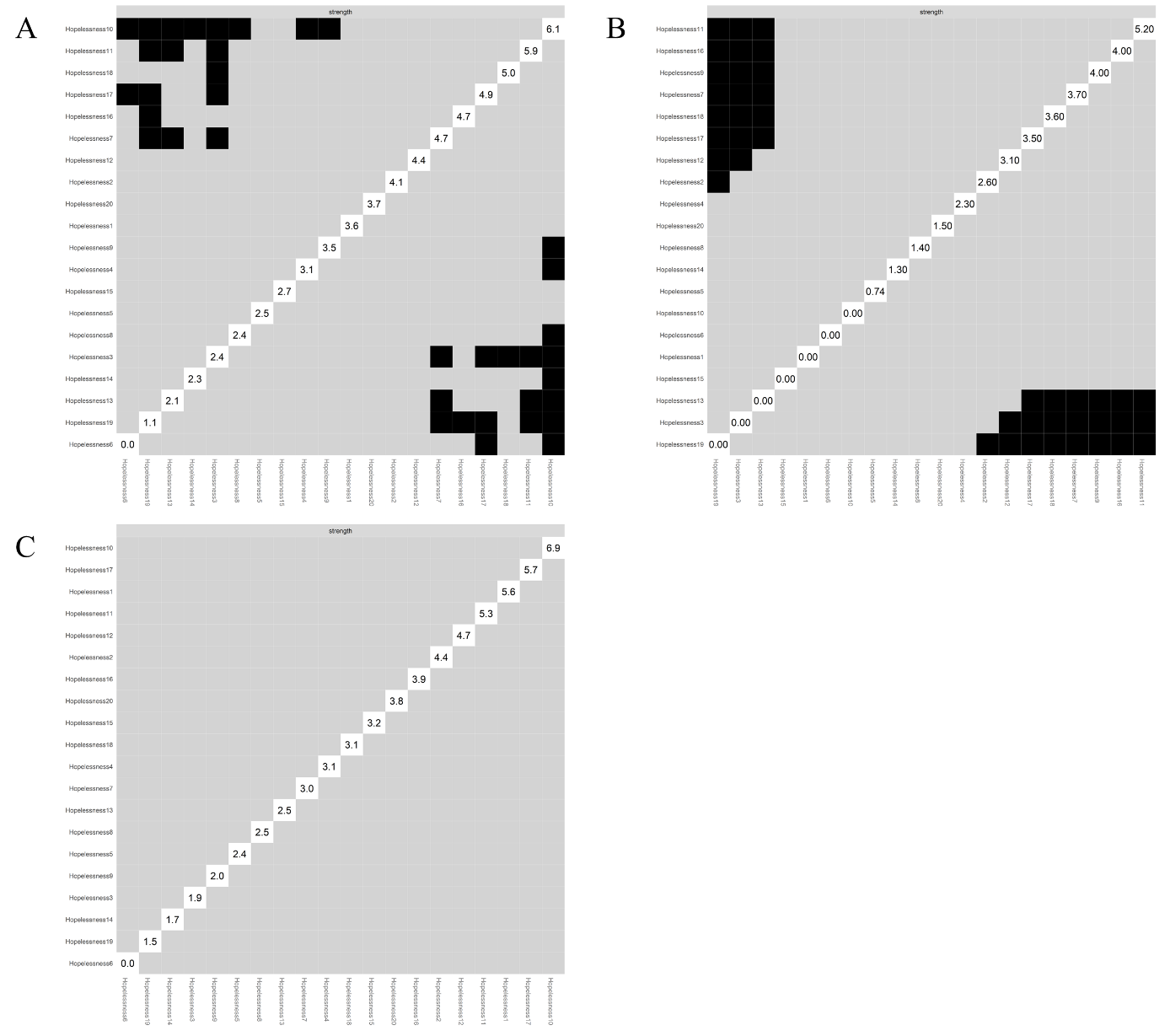


Figure S3. Bootstrapped stability test for edge-weight. The results of the bootstrapped difference tests (α=0.05) for edge-weights were shown in this figure. The colour of the boxes indicates whether edge-weights differ significantly from each other (i.e., black) or do not differ significantly (i.e., grey). A indicates all parents. B indicates fathers. C indicates mothers.


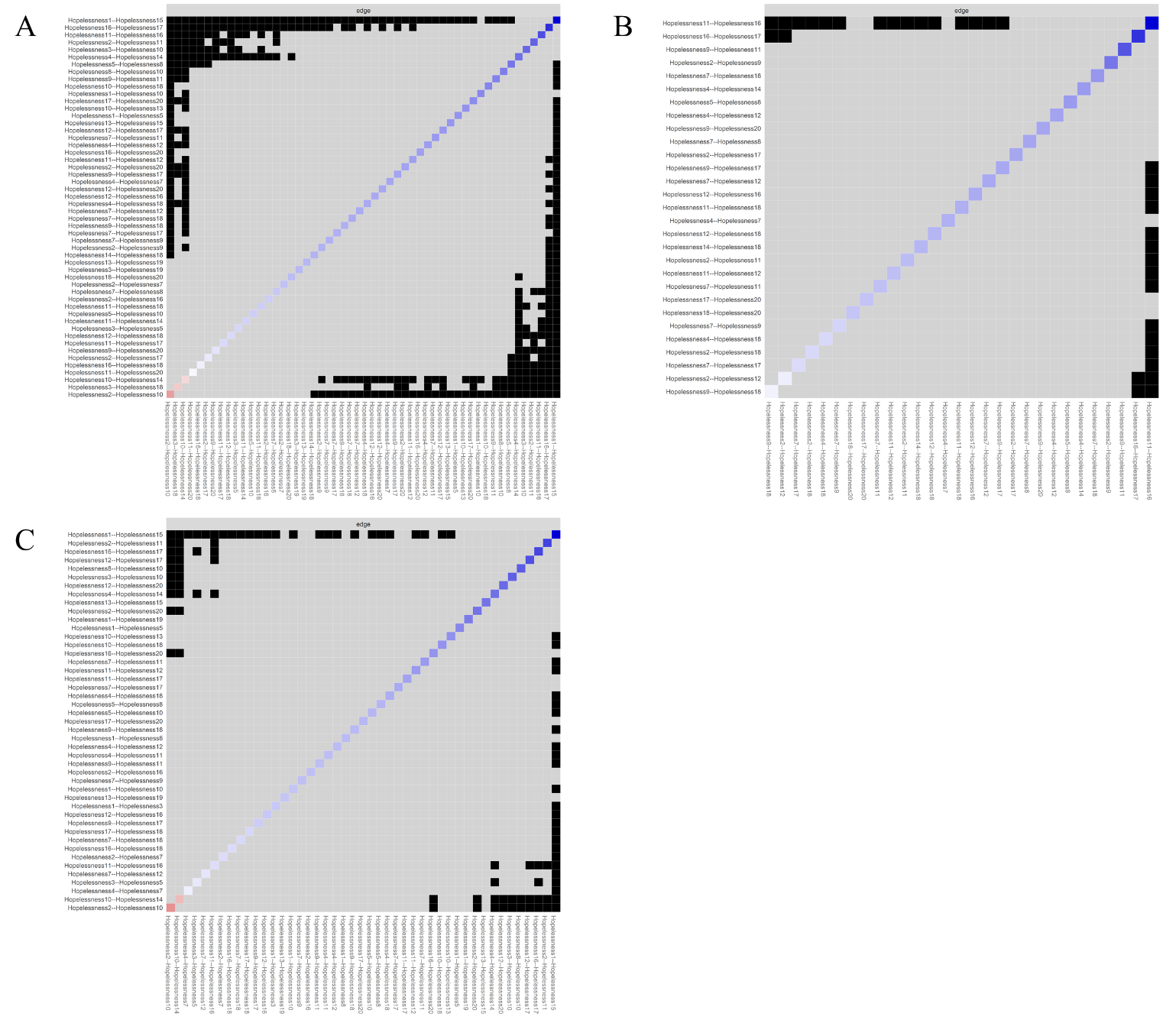


Figure S4. Bootstrapped stability test for edge-weight. The results of the bootstrapped difference tests (α=0.05) for edge-weights were shown in this figure. The colour of the boxes indicates whether edge-weights differ significantly from each other (i.e., black) or do not differ significantly (i.e., grey). The diagonal line indicates the strength of edge-weights, shifting from red (negative associations) to white (representing weaker edges) and ultimately blue (representing stronger edge-weights). A indicates all parents. B indicates fathers. C indicates mothers.
